# Experimental Study on Ultrafine Particle Removal Performance of Portable Air Cleaners with Different Filters in an Office Room

**DOI:** 10.3390/ijerph13010102

**Published:** 2016-01-05

**Authors:** Huan Ma, Henggen Shen, Tiantian Shui, Qing Li, Liuke Zhou

**Affiliations:** School of Environmental Science and Engineering, Donghua University, Shanghai 201620, China; mh00102@126.com (H.M.); 13122427783@163.com (T.S.); saraleeqing@163.com (Q.L.); zhouliu_ke@126.com (L.Z.)

**Keywords:** portable air cleaners, PM_1.0_, single-pass efficiency, effectiveness, electret medium

## Abstract

Size- and time-dependent aerodynamic behaviors of indoor particles, including PM_1.0_, were evaluated in a school office in order to test the performance of air-cleaning devices using different filters. *In-situ* real-time measurements were taken using an optical particle counter. The filtration characteristics of filter media, including single-pass efficiency, volume and effectiveness, were evaluated and analyzed. The electret filter (EE) medium shows better initial removal efficiency than the high efficiency (HE) medium in the 0.3–3.5 μm particle size range, while under the same face velocity, the filtration resistance of the HE medium is several times higher than that of the EE medium. During service life testing, the efficiency of the EE medium decreased to 60% with a total purifying air flow of 25 × 10^4^ m^3^/m^2^. The resistance curve rose slightly before the efficiency reached the bottom, and then increased almost exponentially. The single-pass efficiency of portable air cleaner (PAC) with the pre-filter (PR) or the active carbon granule filter (CF) was relatively poor. While PAC with the pre-filter and the high efficiency filter (PR&HE) showed maximum single-pass efficiency for PM_1.0_ (88.6%), PAC with the HE was the most effective at removing PM_1.0_. The enhancement of PR with HE and electret filters augmented the single-pass efficiency, but lessened the airflow rate and effectiveness. Combined with PR, the decay constant of large-sized particles could be greater than for PACs without PR. Without regard to the lifetime, the electret filters performed better with respect to resource saving and purification improvement. A most penetrating particle size range (MPPS: 0.4–0.65 μm) exists in both HE and electret filters; the MPPS tends to become larger after HE and electret filters are combined with PR. These results serve to provide a better understanding of the indoor particle removal performance of PACs when combined with different kinds of filters in school office buildings.

## 1. Introduction

Several severe haze-fog periods caused by fine particles have been observed in Shanghai in recent years. The greatest increase in particle number concentration during haze events is in the 0.5–1 μm size fractions with levels about 18 times those during non-haze days [1]. Epidemiological and toxicological studies on the influence of indoor air fine particles (of aerodynamic diameters smaller than 2.5 μm) and ultrafine particles (of aerodynamic diameters smaller than 1.0 μm) on respiratory and cardiovascular morbidity and mortality have been conducted [2,3,4]. Ultrafine particles are considered especially hazardous as they can penetrate deep into the respiratory system [5].

Research has found that the average person spends 70%–80% of their time indoors in developed countries [6], and approximately 85% in China [7]. Indoor particle pollution, therefore, can be expected to contribute significantly to any morbidity and mortality. Because of this, the indoor particle levels are a key factor in health assessment and device evaluation studies.

Easily available, applicable, convenient and relatively inexpensive portable air cleaners (PACs) are becoming more and more popular for the control of indoor air particle pollution [8]. Numerous investigations have demonstrated that PACs can significantly reduce indoor particle matter (PM) concentrations [9,10,11]. It has been reported that about 10%–30% of homes are equipped with PACs for improving indoor air quality in developed countries [12]. PACs may provide an effective solution for controlling concentrations of office indoor fine particles. PACs have been widely researched, and various technologies have been used for PACs, including High Efficiency Particulate Air (HEPA) filters, electrostatic precipitators (ESPs), ion generators, composite filters comprised of activated carbon and HEPA filters [12,13,14]. Shaughnessy *et al.* demonstrated that PACs with HEPA filters are more effective in removing indoor particles than electret filter systems, ionizers, and ozone generators with large source chambers [15].

Numerous studies investigating the performance of PACs with HEPA filters and ESPs have already been conducted, and have illustrated that the effectiveness of HEPA filters and ESPs is typically high [10,16,17]. However, most of these studies were conducted using time-consuming measurements; Michael S. *et al.* presented the real-time size-dependent aerodynamic properties of removal devices in a stainless steel chamber [17], while the *in-situ* real-time, size-dependent aerodynamic properties of removal devices are seldom considered.

This study presents an experiment on the particle removal performance of portable air cleaners with different types of filters. Size- and time-dependent aerodynamic behaviors of indoor particles, as well as PM_1.0_, were measured in a school office. The filtration characteristics of filter media and the single-pass efficiency, volume and effectiveness of the PAC were evaluated, and the differences between PACs with and without the active carbon granule filter (CF) and the pre-filter (PR) were investigated. The results of this study aim to provide a better understanding of the indoor particle removal performance of PACs under different kinds of filters in school office buildings.

## 2. Experiments

### 2.1. Facilities and the Tested PAC

**Figure 1:**
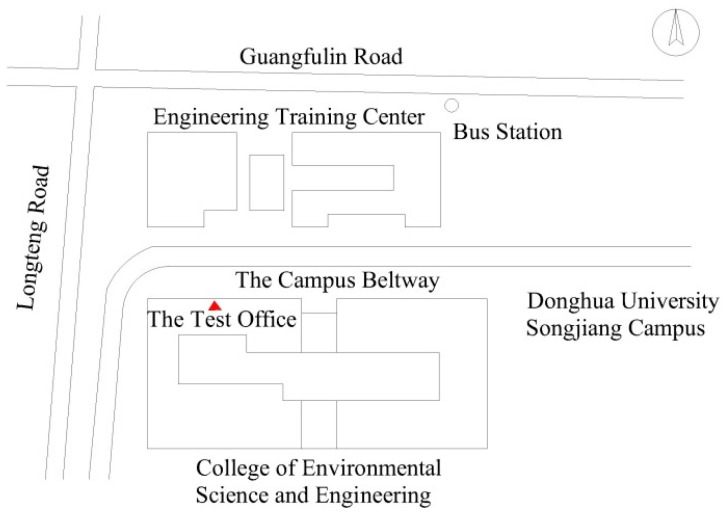
Location of the tested office building.

**Figure 2:**
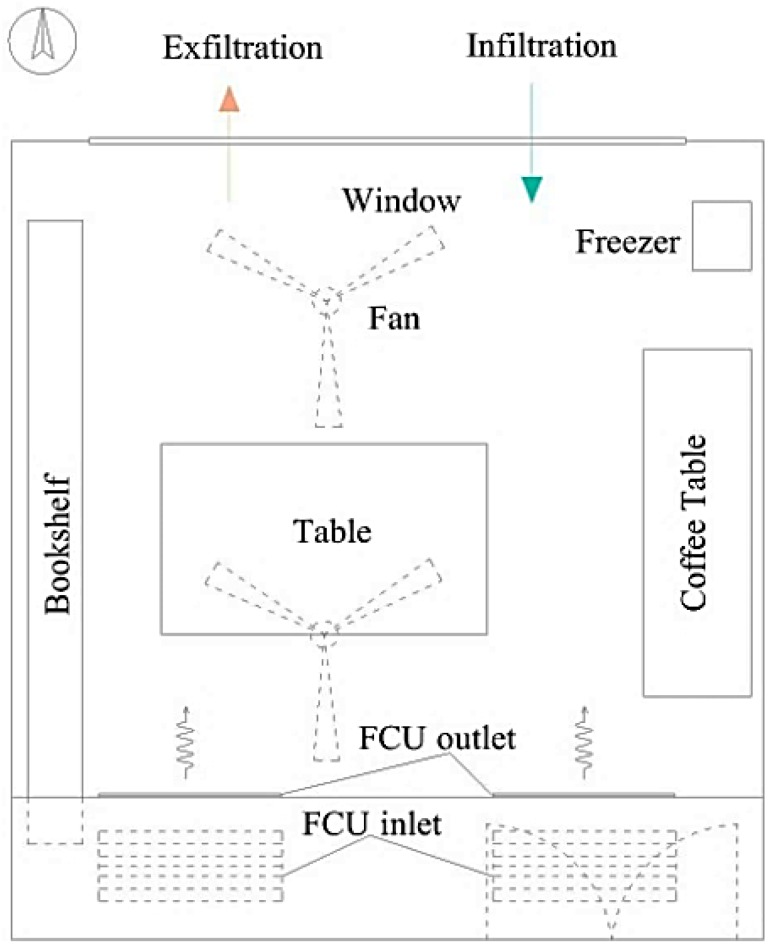
Schematic of the tested office room.

The tested building is located in the southwest corner of Donghua University, indicated by the triangle in Figure 1. The university is in the southwest part of the Songjiang District, Shanghai. The building of interest was built in 2003 and consists of five stories. It is situated near the campus beltway. The tested office room is on the third floor on the northern side (northern hemisphere), and its volume is about 87.5 m^3^ (5.4 m × 6.0 m × 2.7 m). The tested room functions as a meeting room during working hours, and provides a suitable place for staff and students to share their experiences. It has wooden flooring as well as wallpaper. The furniture includes a large bookshelf, a large table, a coffee table, a freezer and a variety of books. The ventilation design in the room is an fan coil unit (FCU), and there is no fresh air system. Fresh air is mainly provided by the infiltration from cracks around windows and doors. This system is common in existing buildings in China, especially in second-tier cities. A detailed schematic of the room and ventilation system is shown in Figure 2. Because the tests were conducted while the room was unoccupied, the occupant density and furniture layout remained constant for the entire study.

**Figure 3:**
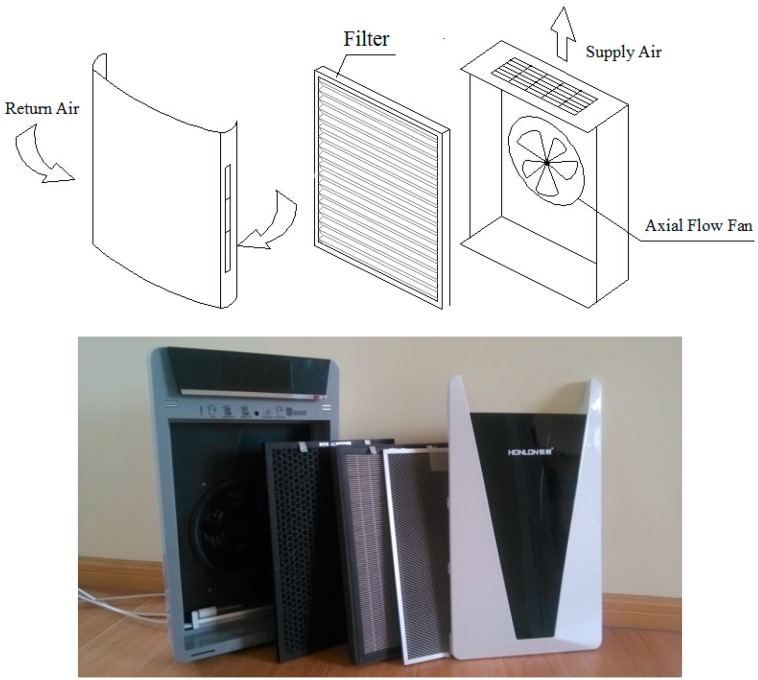
Schematic diagram of the portable air cleaner.

The tested PAC is a HLXK-1A Stand-alone unit which is a domestic brand. The airflow configuration of the selected PAC is a double side return and upside supply, the most common configuration. Detailed information about the tested PAC is shown in Table 1, and a schematic diagram of the PAC is shown in Figure 3, demonstrating that the PAC is equipped with an axial flow fan and two kinds of filters. Tested filters include the original series and a custom-made series. The original series refers to pre-filters (PR), high-efficiency filters (HE) and active carbon filters (CF). Electret filters (EE) are custom made by an original equipment manufactory (OEM), and made of 250 g/m^2^ of electret filtration materials. The detailed characteristics of the filter media are shown in Table 1. All filters are designed with sealing strips to prevent bypass, as shown in Figure 3. The filter changes in the PAC do not change the airflow geometry of the unit.

**Table 1:** Descriptions of the portable air cleaner, filters and filter media tested in this study.

**PAC**		**Description**	
Model		HLXK-1A Stand-alone Unit	
Applied area		40 m^2^–60 m^2^	
Flow type		Double side return and upside supply (Figure 3)	
Air supply W (mm) × H (mm)		145 × 245	
**PAC Filters**			
Normal Filters:	Pre-filter (PR)		W × H × D (mm) 290 × 420 × 45
	High efficiency filter (HE) (1.68 m^2^)		
	MERV16 (>96%)		
	Active carbon granule filter (CF)		
Electret filters: Fold number × Fold interval (mm)	EE6	6 × 45 (0.20 m^2^)	
	EE10	10 × 45 (0.29 m^2^)	
	EE20	20 × 45 (0.54 m^2^)	
**Filter Media**			
	Thickness (mm)	Fiber diameter (μm)	Packing density (%)
HE	0.562 ± 0.006	6.108 ± 0.074	8.548 ± 0.064
EE	3.841 ± 0.010	15.231 ± 0.878	7.029 ± 0.291

### 2.2. Instruments and Measurement Procedures

#### 2.2.1. Filtration Characteristics of Filter Media

**Figure 4:**
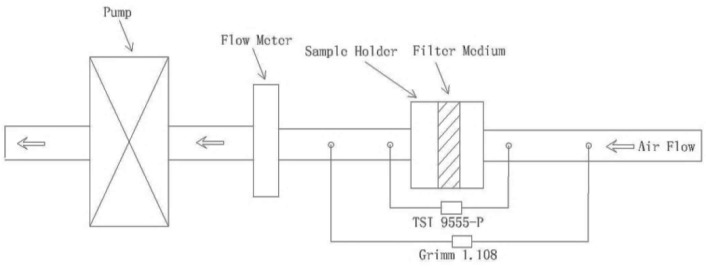
Schematic diagram of the duct system.

The efficiency and pressure drop of the filter media were evaluated and measured using a duct system (Figure 4). The system consisted of a duct, a sample holder, a flow meter, a pump and a pump control. The experiments were carried out in a laboratory of Donghua University. The particle source is environmental particles. The ventilation system was turned off and the windows were closed in the laboratory to keep the particle concentration relatively stable. A Grimm 1.108 “Filter-check™” Submicron Aerosol Spectrometer was used to measure the particle numeric concentration in the duct before and after the filter medium was mounted in the duct. The particle concentration was collected alternatively in the duct before and after the filter medium. The pressure drop was monitored by a TSI 9555-P velocity calculator. The sampling time was set to 5 s for the TSI 9555-P and 6 s for the Grimm 1.108. Detailed information related to the test instruments is listed in Table 2. Each test lasted for a total of 120 s and was repeated twice. Based on the recorded and averaged data, the filtration efficiency was calculated using Equation (1):

(1) $$\eta =\left(1-\frac{N_{supply}}{N_{return}}\right)\times 100\%$$

where $N_{supply}$ is the average PM_1.0_ concentration in the PAC outlet (particles/m³), and $N_{return}$ is the average PM_1.0_ concentration in the PAC inlet (particles/m³).

**Table 2:** Test instruments for filtration characteristics.

Name	Type	Origin	Unit	Sample Flow Rate	Sensitivity	Range
Grimm Sub-Micron Aerosol Spectrometer	1.108	Germany	p/L or μg/m³ (EN 481 or U.S.-EPA)	1.2 L/min	1 p/L or 0.1 μg/L	0.1–100,000 μg/m³ or 1–2,000,000 p/L
Velocity calculator	TSI 9555-P	USA	m/s	-	0.01 m/s or ±1%	0–50 m/s or −3735–+3735 Pa

The service life of the electret filter medium was also tested using the duct system. The filtration efficiency and pressure drop were collected every 8 h after the system was carried out. In order to accelerate the process the test was conducted with a face velocity of 1.0 m/s, nearly 10 to 20 times as high as the filtration velocity of the HE medium, while the filtration efficiency and resistance were collected using a face velocity of 0.1 m/s.

#### 2.2.2. Single-Pass Efficiency and Airflow Rate

The PM_1.0_ numeric concentrations in the PAC inlet and outlet were measured using an Aerosol Spectrometer. The Spectrometer is capable of measuring particles of between 0.3 and 20 μm in diameter. For the purposes of this study, particle counts were divided into different size bins: 0.3–0.4 μm, 0.4–0.5 μm, 0.5–0.65 μm, 0.65–0.8 μm, 0.8–1.0 μm, 1.0–2.0 μm, 2.0–3.0 μm, 3.0–4.0 μm, 4.0–5.0 μm, 5.0–7.5 μm, 7.5–10.0 μm and 10.0–15.0 μm. The air supply velocity was measured using the TSI 9555-P velocity calculator. The tests were conducted in the test room. The window was close and the ventilation system was turned off to ensure that the airflow was not disturbed. The particle concentrations of inlet and outlet were collected alternatively. There was no ventilation and no particle sources in the test room and the concentrations at the inlet and outlet were relatively stable. Each test continued for a total of 120 s and was repeated twice. From the collected and averaged data, the single-pass efficiency and airflow rate of the PACs with different filters were calculated using Equations (1) and (2):

(2) $$Q=v\times \;A_{s}\times 3600$$

where v is the average air supply velocity (m/s), and $\;A_{s}$ is the area of the air supply outlet (m^2^).

#### 2.2.3. Effectiveness

**Figure 5:**
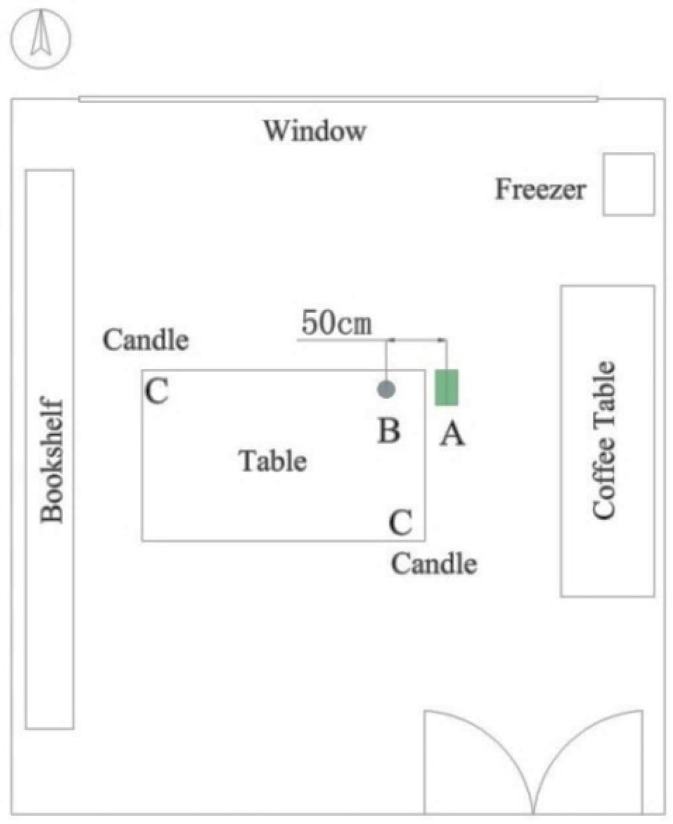
Schematic drawing of the experimental setup.

The experimental setup for the measurements of indoor real-time fine-particle behavior is shown in Figure 5. Indoor real-time fine-particle concentrations were also monitored using a Grimm 1.108 aerosol spectrometer. The aerosol spectrometer probe was located at point B in the breathing zone of the sitting people (1.5 m), as shown in Figure 5. In order to keep the initial indoor particle concentration of each test at the same level, two pure wax candles were burned at points C and D for 20 min before each test. In order to have sooting flames, two circulation fans were turned on. When the concentration of candle smoke particles reached a relatively constant value, the circulation fans were turned off, the PAC, which was at location A, was turned on, and particle concentration data were acquired at 1 min intervals for a duration of 60 min. Each filter condition was performed three times under the same experimental condition. The initial indoor fine particle concentration in each test was kept constant at (0.9–1.1) × 10^9^ particles/m³. The ventilation system was shut down and the windows were closed during the tests. There was no other particle source during the tests.

The effectiveness of the PAC with different filters, as collected by the aerosol spectrometer, is expressed as the real-time particle decay constant, based on the single zone mass balanced model derived from Hayes [18]. Another particle removal performance metric is the normalized indoor particle concentration, which can be calculated using the following formula:

*C* = *N_t_*/*N_0_* = e*^−kt^* (3)

Thus, the formula for the real-time particle decay constant *k*, the linear regression of *N_t_* and *N*_0_, is given by:

(4) $$k=\frac{\sum_{i}\left(t_{i}\cdot \ln\frac{N_{0}}{Nt}\right)}{\sum_{i}t_{i}^{2}}$$

where *N_t_* is the numeric concentration at time *t_i_* (particles/m^3^), *N*_0_ is the initial concentration at time *t*_0_ (particles/m^3^), *k* is the decay constant (min^−1^), *t* is the time (min), and *C* is the normalized indoor particle concentration (*N_t_*/*N*_0_).

All instruments were tested and calibrated in the laboratory before being used.

### 2.3. Indoor Particle Source

In China, the filtration systems of most public buildings have not yet had much attention paid to them. Most median low structures allow for natural ventilation. So, besides the large particles generated from human activity, the fine particles and ultra-fine particles from the outdoors were also an important particle source, especially during haze-fog events. The average number concentrations of particles in different size fractions on haze and non-haze days are listed in Table 3. It indicates that the greatest increase in particle number concentration is in the 0.5–1 μm size fraction during haze events, about 18 times that found on non-haze days.

**Table 3:** Average number concentration of particles in different size fractions on haze and non-haze days (unit/cm^3^).

Size	10–20 nm	20–50 nm	50–100 nm	0.1–0.2 μm	0.2–0.5 μm	0.5–1 μm	1–10 μm	10 nm–10 μm
Haze	1665	6591	4702	2678	1030	114	14	16,797
Non-haze	1622	3580	1389	631	317	6	2	7547

In order to study the effectiveness of PACs for PM_1.0_, pure wax candles were selected as the particle source. The particles generated from the wax candles span a wide range of particle sizes, namely ultrafine, fine and coarse [5]. Reference [19] pointed out that a sooting flame produces much higher fine particle mass emission rates than a stable burning candle and it emits vast amounts of fine black elemental carbon particles. A sooting flame is usually caused by forced flow. Table 4 shows the mass and count percentage of different sooting smoke particle sizes. The PM_1.0_ in a pure wax candle with the sooting smoke accounts for nearly 98% of the total particle number concentration. Even though there are limitations from the point of view of particle size in choosing candle smoke as the particle source, for the situation in China, it was considered to be a suitable particle source for this study.

**Table 4:** Mass and count percentage of different particle sizes in pure wax candle smoke.

Particle Size (μm)	Percentage of Different Particle Size		SD	
	Mass Percentage	Number Percentage	Mass (μg/m^3^)	Number (P/m^3^)
0.23–0.3	0.3247	-	1.3848	-
0.3–0.4	0.2769	0.7156	1.3532	2,152,291.4469
0.4–0.5	0.1074	0.1700	0.7371	264,275.1092
0.5–0.65	0.1032	0.0794	0.8764	92,065.1943
0.65–0.8	0.0510	0.0211	0.3725	218,006.8806
0.8–1.0	0.0344	0.0094	0.1794	43,588.9894
1.0–1.6	0.0245	0.0027	0.2887	42,720.0187
1.6–2.0	0.0209	0.0010	0.3755	38,157.5681
2.0–3.0	0.0297	0.0007	0.7068	4041.4519
3.0–4.0	0.0063	0.0001	0.2118	2000.0000
4.0–5.0	0.0058	0.0000	0.4353	1154.7005
5.0–7.5	0.0085	0.0000	0.5056	150.0000
7.5–10.0	0.0028	0.0000	0.5744	50.0000
10.0–15.0	0.0043	0.0000	1.4271	16.0728

## 3. Results and Discussion

### 3.1. Filtration Characteristics of Filter Media

**Figure 6:**
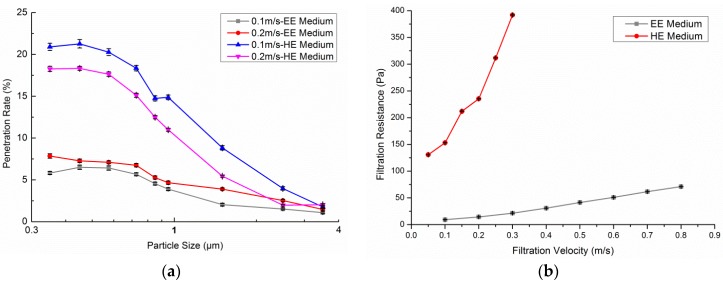
Filtration characteristics of clean EE and HE medium. (**a**) Size resolved penetration rate; (**b**) filtration resistance.

The penetration rates for particles of different sizes and filtration resistance for different face velocities for clean EE and HE media are illustrated in Figure 6. For clean medium, the penetration rate of very fine particles increases with increasing flow velocity because a higher velocity leads to a higher driving force, which may cause more particles to pass through the open channels. As can be seen from the graph, the EE medium shows better removal efficiency than the HE medium in the 0.3–3.5 μm range, especially for particles beyond the 1 μm size. With a 0.2 m/s face velocity, the penetration rate of clean HE medium commences at 20% in the 0.3–0.6 μm range, and reduces to 3% in the 3–4 μm range. While the penetration rate for clean EE medium is only 7.5% in the 0.3–0.6 μm range, under the same face velocity, it reached 3% in the 3–4 μm range as well.

The increase in face velocity brings about high drag forces, leading to an increase in filtration resistance. As can be seen in Figure 6, under the same face velocity, the filtration resistance of the HE medium is several times that of the EE medium, and the increasing rate of the HE medium is also several times that of the EE medium.

**Figure 7:**
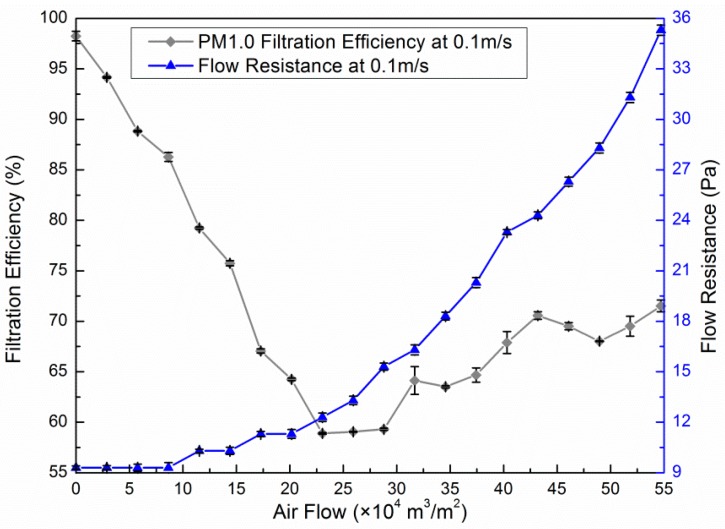
PM_1.0_ efficiency and pressure drop curves of an electret filter medium.

Figure 7 shows PM_1.0_ efficiency and pressure drop curves according to the blow rate of an electret filter medium with a filtration velocity of 0.1 m/s. Particle loading generally increases the collection efficiency as well as the pressure drop of the filter media in the absence of electrostatic forces. In contrast, the dendrites that appeared in dust loading increase the penetration rate of the filter media made of charged fibers in the early stages of filtration since the deposited particles diminish electrostatic effects [20]. As shown in Figure 7, the initial PM_1.0_ efficiency was 98% when the medium was clean, decreasing to a minimum efficiency of about 60% with a total purifying air flow of 25 × 10^4^ m^3^/m^2^ (nearly equal to 90 days under a condition of 0.1 m/s and 8 h/day). Then, the efficiency increased slowly to 70% and the deposited particles began to form a cake. The resistance curve rose slightly before the efficiency reached the bottom and then augmented almost exponentially with increasing total purifying air flow. The results are consistent with those of Walsh D.C. [21].

### 3.2. Single-Pass Efficiency for PM_1.0_ and Airflow Rate

**Figure 8:**
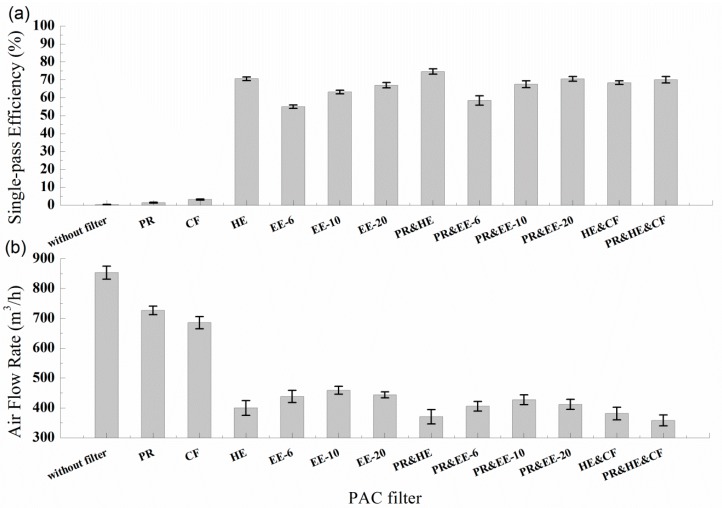
Single-pass efficiency and airflow rate of PACs with different filters. (**a**) Single-pass Efficiency; (**b**) Air Flow Rate.

Single-pass efficiency and airflow rate are two major parameters for estimating the effectiveness of air clean devices. The single-pass efficiency for PM_1.0_ and the airflow rate of PACs with six single filters and six multiple filters, which were calculated using Equations (1) and (2), are shown in Figure 8a,b. The results indicated that PR and CF both had a single-pass efficiency of less than 3%. When coagulation and natural decay were taken into consideration, the PR and CF were shown to have hardly any effect on the efficiency of the PAC. PR&HE had the best single-pass efficiency, which was 74.6%. At the same time, the airflow rate of PR&HE was not the lowest, and was still a little higher than the PR&HE&CF and HE&CF filters. The single-pass efficiency of PR&HE&CF was 70.0%, 4.6% lower than for PR&HE. Uniformly, when combined with CF, the single-pass efficiency of the HE decreased from 70.6% to 68.4%. CF has been used widely for removing harmful gaseous pollutants [22]. However, these results show that CFs may generate air resistance increases and lead to a bypass of the HF. PR and electret filters were shown to be more effective. For example, the single-pass efficiency of HE combined with PR was 74.6%, as opposed to 70.6% with HE alone.

The single-pass efficiency of electret filters in Figure 8a was shown to be positively linked to the filter area. The airflow rate of electret filters in Figure 8b fell after first increasing, along with a raise in the filter area: EE-6: 438.3 m^3^/h, EE-10: 459.1 m^3^/h, EE-20: 443.8 m^3^/h. This is because the filtration resistance mainly includes a pressure drop from the filtration medium and filter geometry [23].

### 3.3. Natural Decay

**Figure 9:**
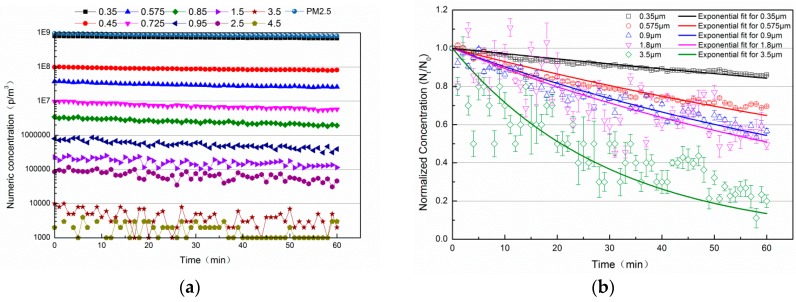
Numeric and normalized concentration over time of indoor particles with different particle sizes with natural decay. (**a**) Numeric concentration; (**b**) Normalized concentration.

It is relatively simple to measure the natural decay rate coefficient by monitoring the decrease in the particle concentration over time without the use of a PAC. Figure 9 documents the changes in numeric concentrations of indoor particles as they undergo natural decay. These results illustrate that without the operation of a filtration system, when the decay in the concentration of indoor particles was only due to gravitational settling, the initial concentrations of the different particle sizes can vary by several orders of magnitude. Figure 9b shows the normalized concentration and the corresponding fitting results. Moreover, the natural decay constant over 1 h and the corresponding residual sum of squares based on these fitting results are illustrated in Table 5. The concentration decay constant of particles of 0.35 μm and 3.5 μm were approximately 0.2450 and 2.388 over one hour, respectively. After 1 h, the concentration of particles of five different sizes (0.35 μm, 0.575 μm, 0.9 μm, 1.8 μm, 3.5 μm) decreased to 0.85, 0.65, 0.55, 0.50 and 0.12, respectively. These results indicate that the concentration of particles with larger sizes decreased the most quickly because of the effects of gravitational settling.

**Table 5:** The natural decay constant over 1 h of particles with various particle sizes and the corresponding residual sum of squares.

Particle Sizes	0.35 μm		0.575 μm		0.85 μm		1.8 μm		3.5 μm	
	Value × 10^−2^	RSS/dof × 10^−3^	Value × 10^−2^	RSS/dof × 10^−3^	Value × 10^−2^	RSS/dof × 10^−3^	Value × 10^−2^	RSS/dof × 10^−3^	Value × 10^−2^	RSS/dof × 10^−3^
Natural decay	0.2450	0.1191	0.3020	0.1450	0.2130	1.130	0.6130	5.640	2.388	11.16

### 3.4. Effectiveness

#### 3.4.1. Effectiveness for Removing Indoor PM_1.0_

**Figure 10:**
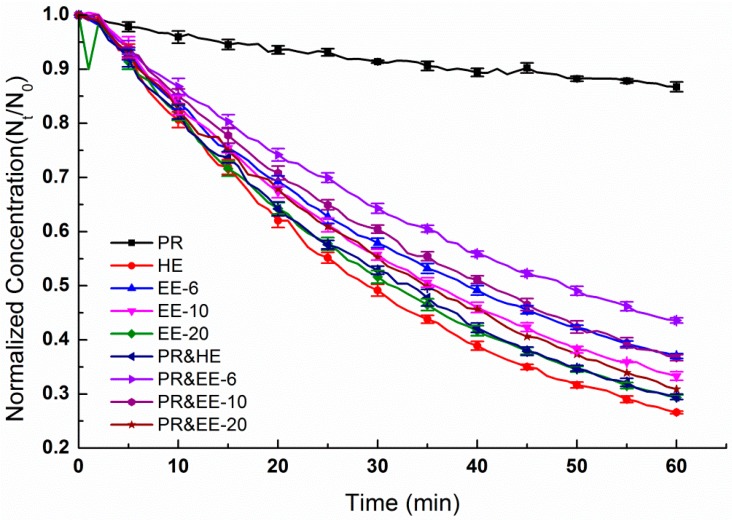
Numeric concentration over time of indoor PM_1.0_ with PAC with different filters.

Figure 10 compares the numeric concentrations of indoor PM_1.0_ as the PAC operated over one hour with a variety of filter types. Using PACs with HE, EE-6, EE-10, and EE-20 filters greatly reduced PM_1.0_ exposures, while CF was relatively ineffective and reduced particle concentrations only slightly. After 1 h, the normalized PM_1.0_ concentration of the PAC with five kinds of filters (PR, HE, EE-6, EE-10, and EE-20) dropped below 13.3%, 73.4%, 63.0%, 66.7% and 70.6%, respectively (Figure 10). As shown in Table 1, the filter areas of HE, EE-6, EE-10 and EE-20 were 1.68 m^2^, 0.20 m^2^, 0.29 m^2^ and 0.54 m^2^. The filter area of the HE was 2–7 times larger than the electret filters, while the percentage of attenuation of the HE was only 2%–10% larger than that of the electret filters. As for the electret filters with different medium areas, the percentage of attenuation over one hour improved 7% when the filter area was 1.7 times larger. Therefore, without regard to the lifetime, electret filters were shown to perform better in the areas of resource saving and purification improvement.

When HE, EE-6, EE-10 and EE-20 were coupled with PR, the percentage of attenuation over one hour was reduced by 2.9%, 6.6%, 3.4% and 1.5%, respectively. Regarding PACs, current air-cleaning techniques include high efficiency particulate air filtering (HEPA), adsorption, ultraviolet germicidal irradiation (UVGI), photo catalytic oxidation (PCO), thermal catalytic oxidation (TCO), plasma, botanic air cleaners, ion generators, and electrostatic precipitators [14]. The results of this study actually indicate that an increase in filter layers does not necessarily precipitate an increase in PAC effectiveness.

In order to investigate the reason for this reduction in effectiveness, a single zone mass balanced model [24] without the use of a central air conditioning (HVAC) system in the residences to estimate indoor levels of particles is cited here (Equation (5)). This model works well in the evaluation of indoor particle counting concentrations of air conditioning filtration systems, surface deposition and coagulation [25]. Under the assumption that chemical reactions are negligible and that the pollutants are well mixed [26,27], the model can be simulated using the ventilation parameters, indoor initial particle concentrations, outdoor particle concentrations and indoor particle source characters.

(5) $$N_{in}^{t_{i}}=N_{in}^{t_{i-1}}e^{-\frac{3600\varepsilon }{V}\left(\eta Q+Q_{exf}+Vk_{n}\right)\Delta t}+\frac{3600}{V}N_{out}^{t_{i}}Q_{exf}\Delta t(\mathrm{particles}/m^{3})$$

where 𝜀 is the mixing factor (unitless; assume that 𝜀 = 1 refers to perfect air mixing conditions), $k_{n}$ is the natural decay rate of PM_1.0_ at time $t_{i}$ (*s^−1^*), Δt is the time period (*h^−1^*), $Q_{exf}$ is the air exchange flow rate from the building crack (m^3^/s), and $N_{out}^{t_{i}}$ is the outdoor PM_1.0_ concentration at time $t_{i}$ (particles/m^3^).

The ventilation rate of the PAC with different filters ranges from 5 to 10 in this study. So, in the tests, it is assumed that the conditions are well mixed, and ηQ is the key factor that influences the indoor particle concentration over time under the conditions of this study. In previous studies [28], the clean air delivery rate (CADR) has been defined as the product of the single-pass efficiency and the airflow rate (ηQ).

**Figure 11:**
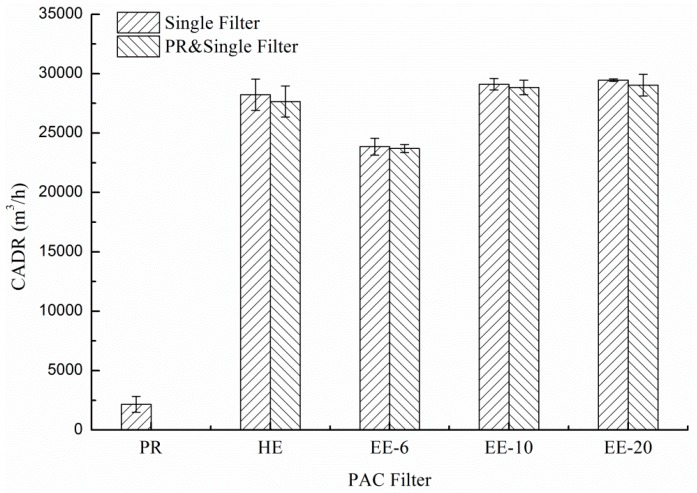
CADRs with and without PR filter, according to single filter.

Based on the tests results regarding single-pass efficiency and airflow rate, ηQ values of four kinds of PACs with and without PR filters are shown in Figure 11. When HE, EE-6, EE-10 and EE-20 were coupled with PR, the CADRs reduced from 282.2 m^3^/h, 238.5 m^3^/h, 291.1 m^3^/h and 294.5 m^3^/h to 276.5 m^3^/h, 236.9 m^3^/h, 288.4 m^3^/h and 290.3 m^3^/h, respectively. Therefore, the percentage of attenuation over one hour was reduced after filters were coupled with PR.

#### 3.4.2. Effectiveness for Removing Particles of Different Diameters

**Figure 12:**
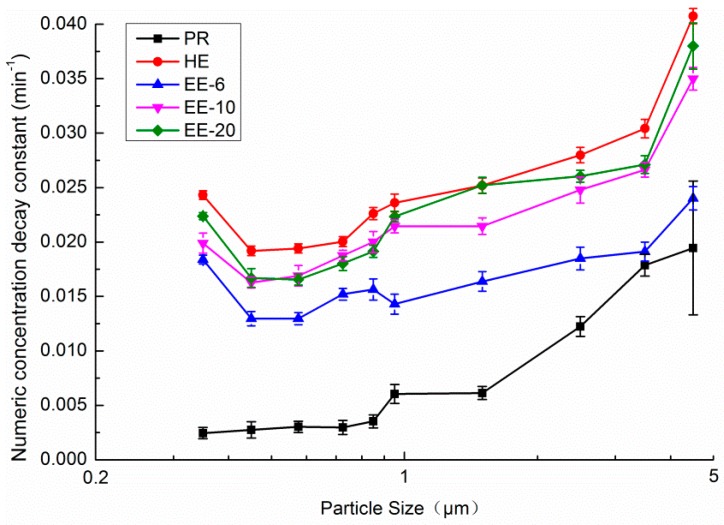
Size-resolved numeric concentration decay constant over 1 h for PACs with different single filters.

Figure 12 displays the size-resolved numeric concentration decay constant over 1 h for PACs with different single filters. CF filters proved to be relatively ineffective for particles of less than 1 μm, and for particles larger than 1 μm, the decay constants improved from 0.006 to 0.020 as the particle size increased. A most penetrating particle size (MPPS) region exists in both HE and electret filters. In this case, the MPSS was 0.4–0.65 μm. The decay constant is at a minimum in this region. This phenomenon can be explained by the fact that under the same circumstances, the decay constant decreases with a reduction in the single-pass efficiency. In the meantime, a MPPS region for fibrous filter medium exists [29]. Besides the single-pass efficiency, the natural decay constant and the outdoor concentration are also a function of particle size.

**Figure 13:**
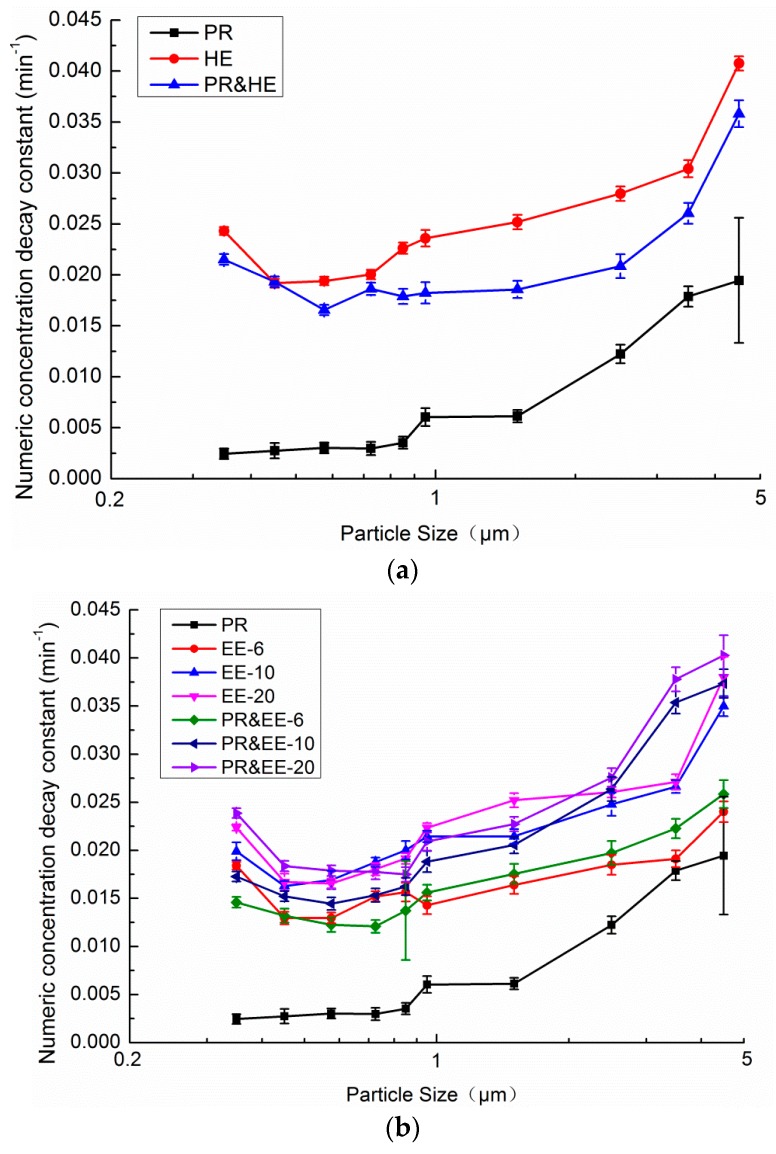
Size-resolved numeric concentration decay constant over 1 h for single filter PACs with and without PR filter. (**a**) High efficiency filter; (**b**) Electret filters.

Figure 13 shows the size-resolved numeric concentration decay constant over 1 h for single filter PACs with and without PR filters. Figure 13a shows that the decay constant of HE decreased slightly after it was combined with PR. Figure 13b shows that the decay constant of electret filters also decreased slightly when combined with PR for very small size particles, while the decay constant increased significantly for particles of larger sizes. At a specific particle size, the decay constant with PR was equal to the condition without PR. In the cases of EE-6, EE-10 and EE-20, the specific particle sizes were 0.95 μm, 1.8 μm and 2.1 μm, respectively. The major reason for the changes in the decay constant after combining with PR could be the influence of the combination with PR on CADR decreasing with the reduction of the single-pass efficiency. Figure 11 demonstrates that the CADR loss after the filter was combined with PR is 571.1 m^3^/h, 160.6 m^3^/h, 267.1 m^3^/h, and 415.7 m^3^/h for HE, EE-6, EE-10, and EE-20, respectively. For small particles, the increase in single-pass efficiency cannot make up for the influence of the reductions to the airflow rate. For large-sized particles, the single-pass efficiency of the filters increased significantly as the particle size increased. Therefore, the decay constant with PR can be equal to the constant without PR at a specific particle size (0.95 μm, 1.8 μm and 2.1 μm for EE-6, EE-10 and EE-20 filters, respectively). The MPSS was found to increase when both the HE and electret filters were combined with PR.

## 4. Conclusions

The effect of different filters on the particle removal performance of portable air cleaners was investigated experimentally. The single-pass efficiency, airflow rate, and time- and size-dependent particle removal performance for PACs with different kinds of filters were examined. The conclusions of this study are summarized below:

• Filtration characteristics of filter media: A clean EE medium shows better removal efficiency than a clean HE medium in the 0.3–3.5 μm range, especially for particles above 1 μm in size. Under the same face velocity, the filtration resistance of the HE medium is several times more than for the EE medium, as well as demonstrating an increasing rate. During the tests on the service life of the EE medium, the PM_1.0_ efficiency decreased by about 38% to 60% with a total purifying air flow 25 × 10^4^ m^3^/m^2^. Then, the efficiency increased slowly to 70% and the deposited particles began to form a cake. The resistance curve rose slightly before the efficiency reached its minimum and then increased almost exponentially with increasing total purifying air flow.
• Single-pass efficiency for PM_1.0_ and airflow rate: The single-pass efficiency of PR and CF are relatively ineffective. PR&HE had the maximum single-pass efficiency for PM_1.0_ (88.6%). The enhancement of PR with HE and electret filters augments the efficiency, but lessens the airflow rate. When filters were combined with CF, their efficiency and airflow rates were both reduced. Therefore, it is recommended that the CF be equipped over the other filters to minimize the negative influence.
• Effectiveness for removing indoor PM_1.0_: HE proved to be the most effective filter. Without regard to the lifetime, electret filters performed better in terms of resource saving and purification improvement. The percentage attenuation over one hour was reduced after filters were combined with PR. This shows that filter layer augmentation does not always improve the effectiveness of PACs.
• Effectiveness for removing particles with different diameter sizes: The effectiveness of PR became distinct when particles were larger than 1 μm. A most penetrating particle size (MPPS) region exists in both HE and electret filters. In this case, the MPPS was 0.4–0.65 μm. The MPPS tended to become larger after filters were combined with PR for both HE and electret filters. Combined with PR, the decay constant of large size particles could be larger than the PACs without PR.
• The influence of the mixing factor is ignored in this study. More investigations and laboratory measurements are still needed to explain the effect of room airflow patterns on the spatial distribution of particles.
